# Systematic identification and characterization of cardiac long intergenic noncoding RNAs in zebrafish

**DOI:** 10.1038/s41598-017-00823-3

**Published:** 2017-04-28

**Authors:** Lei Wang, Xiao Ma, Xiaolei Xu, Yuji Zhang

**Affiliations:** 1 Department of Epidemiology & Public Health, University of Maryland School of Medicine, Baltimore, MD 21201 USA; 2 Division of Biostatistics and Bioinformatics, University of Maryland Marlene and Stewart Greenebaum Comprehensive Cancer Center, Baltimore, MD 21201 USA; 3 Department of Biochemistry and Molecular Biology, Mayo Clinic, Rochester, MN 55905 USA; 4 Division of Cardiovascular Diseases, Mayo Clinic, Rochester, MN 55905 USA; 5 Clinical and Translational Science Track, Mayo Graduate School, Mayo Clinic College of Medicine, Rochester, MN 55905 USA

## Abstract

Long intergenic noncoding RNAs (lincRNAs) are increasingly recognized as potential key regulators of heart development and related diseases, but their identities and functions remain elusive. In this study, we sought to identify and characterize the cardiac lincRNA transcriptome in the experimentally accessible zebrafish model by integrating bioinformatics analysis with experimental validation. By conducting genome-wide RNA sequencing profiling of zebrafish embryonic hearts, adult hearts, and adult muscle, we generated a high-confidence set of 813 cardiac lincRNA transcripts, 423 of which are novel. Among these lincRNAs, 564 are expressed in the embryonic heart, and 730 are expressed in the adult heart, including 2 novel lincRNAs, TCONS_00000891 and TCONS_00028686, which exhibit cardiac-enriched expression patterns in adult heart. Using a method similar to a fetal gene program, we identified 51 lincRNAs with differential expression patterns between embryonic and adult hearts, among which TCONS_00009015 responded to doxorubicin-induced cardiac stress. In summary, our genome-wide systematic identification and characterization of cardiac lincRNAs lays the foundation for future studies in this vertebrate model to elucidate crucial roles for cardiac lincRNAs during heart development and cardiac diseases.

## Introduction

Cardiovascular development is a dynamic, spatiotemporally coordinated, and transcriptionally regulated process involving thousands of genes^1^. Even subtle perturbations of this process can lead to a spectrum of heart diseases, such as congenital heart defects^2^, coronary artery disease^3, 4^, cardiac hypertrophy^5, 6^, and arrhythmias^7^. Traditional laboratory studies have focused on protein-coding genes and their regulatory roles in heart development^8, 9^, but these genes account for less than 3% of the human genome. Recent applications of high-throughput sequencing technologies to analyze the transcriptome in human and other model organisms have revealed a wide spectrum of transcripts that do not encode proteins: the so-called noncoding RNAs (ncRNAs)^10, 11^. Several consortium projects, such as the Encyclopedia of DNA Elements (ENCODE)^12^ and Functional Annotation of the Mammalian Genome (FANTOM)^13^ have reported that ncRNAs substantially outnumber protein-coding genes and perform various specific roles in controlling gene expression and function^14^. Thus, these ncRNAs have emerged as new key players in the cardiovascular regulatory network^15^. Based on the size of their mature transcripts, ncRNAs can be broadly classified as small (<200 nucleotide [nt]) RNAs, large (>200 nt) ncRNAs (lncRNAs), and regulatory RNAs (e.g., rRNA and tRNA). As the largest group of mammalian ncRNA transcripts^16^, lncRNAs have been shown to exert nonredundant roles in diverse biological processes, including chromosome X inactivation, imprinting, splicing, and transcriptional regulation^17–19^. To complete the cardiac transcriptome landscape, investigating the biological roles of lncRNAs during cardiac-related biological processes in a systematic genome-wide manner is essential.

Recent cardiovascular mechanism studies have detected and characterized the unique expression of several lncRNAs under both normal physiological conditions and disease states^20–25^, and the results suggest that lncRNAs substantially affect gene regulation in cardiovascular-related biological processes. For instance, *chast* was shown to promote cardiac remodeling and influence cardiomyocyte hypertrophy^26^, whereas *CARMEN* is a super enhancer–associated lncRNA controlling cardiac specification, differentiation, and homeostasis^27^. Long intergenic noncoding RNAs (lincRNAs), a subclass of lncRNAs that do not overlap with annotated coding regions^28^, are especially interesting because their specific genomic locations facilitate experimental manipulation and computational analysis. LincRNAs have been shown to govern cardiac development and stress responses^22, 23^, to orchestrate cardiac transcriptional programs^29^, and to serve as potential biomarkers and novel therapeutic targets for cardiovascular diseases^25^. For instance, both *Bvht* and *Fendrr* interact with the epigenetic silencing complex polycomb repressive complex 2 (PRC2) to regulate key genes during early cardiac development^22, 23^. However, because comprehensive lincRNA annotation is lacking, only a few lincRNAs have been studied in the context of heart development and cardiac diseases^30^. Existing annotations of lincRNAs in human and other model organisms are usually derived from large-scale sequencing studies^31^. Such annotations, however, may miss lincRNAs that are only expressed during narrow spatiotemporal developmental windows^32–34^. To better understand the potential roles of cardiac lincRNAs (i.e., lincRNAs expressed during heart development), performing genome-wide identification, characterization, and analysis of lincRNAs in the context of heart tissue is necessary.

In this study, we sought to systematically identify and characterize cardiac lincRNAs using the zebrafish model. Specifically, we focused on lincRNAs that are expressed in embryonic and adult hearts. First, we reconstructed *de novo* most of the annotated zebrafish genes in the RefSeq and Ensembl databases. Through an integrative bioinformatics filtering approach, we defined a high-confidence set of 813 cardiac lincRNA transcripts, 423 of which were novel. Both *cis*- and *trans*-level analyses suggested that several cardiac lincRNAs were potentially functionally associated with heart development and cardiac diseases. Independent experimental validation also revealed unique activation patterns of the novel cardiac lincRNA TCONS_00009015 in the doxorubicin (DOX)-induced cardiomyopathy model. Our investigation of cardiac lincRNAs in a vertebrate developmental model revealed that these cardiac lincRNAs have crucial biological roles during heart development and may serve as potential key regulators in biological processes closely related to heart diseases. Our cardiac lincRNA catalog will facilitate future experimental and computational studies to uncover lincRNA functions in heart-associated biological processes and disease mechanisms.

## Results

### Assembly of a high-confidence transcriptome in zebrafish

**Figure 1:**
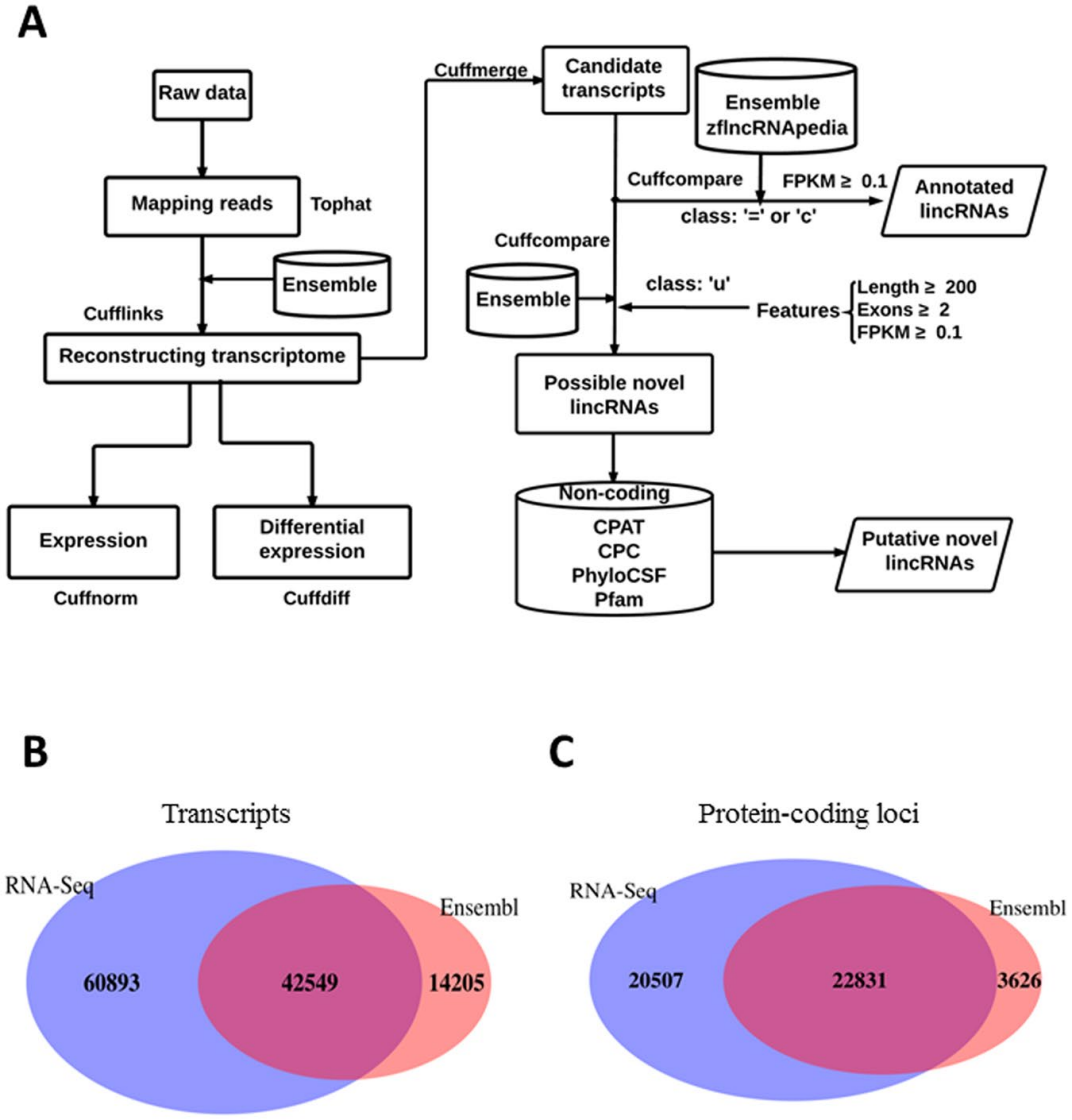
Overview of the RNA-seq–based cardiac transcriptome assembly. (**A**) The computational bioinformatics workflow to assemble the cardiac transcriptome and identify lincRNAs. ‘u’, intergenic transcript in Cufflinks; ‘=’, complete match of intron chain in Cufflinks (see Methods). (**B**) Overlap between transcripts from the RNA-seq–based cardiac transcriptome assembly (blue) and Ensembl annotated transcripts (red). (**C**) Overlap between RNA-seq–based transcripts in protein-coding loci (blue) and Ensembl annotated protein-coding transcripts (red).

Based on our genome-wide cDNA sequencing experiments in 3 tissue types (embryonic heart, adult heart, and adult muscle), which were described in our previous work^35^, we obtained ≈318 million reads. More than 75% of these reads passed the initial quality control step and were aligned to the zebrafish reference genome (Zv9). By applying our stepwise transcriptome assembly pipeline (Fig. 1A), we reconstructed 103,442 transcripts from 43,338 loci across the 3 tissue types; of these transcripts, 87,669 had at least 2 exons, and 15,773 had 1 exon.

To assess the quality and coverage of our reconstructed zebrafish transcriptome, we compared it to the RefSeq (Zv9) and Ensembl annotations (Danio_rerio.Zv9.79). Of 16,146 zebrafish RefSeq transcripts, 12,582 mRNAs (78%) were identified in our zebrafish transcriptome. Similarly, evidence for transcripts from 42,549 of 56,754 Ensembl genes (75%) with comparable transcript structures was identified in our cardiac transcriptome (Fig. 1B). Notably, 22,831 protein-coding genes (86%) in the Ensembl annotation were recovered in our transcriptome (Fig. 1C). In addition, 26,300 transcripts in our zebrafish transcriptome are variants of known Ensembl genes (i.e., novel isoforms or partial transcripts). This high degree of overlap provides independent confirmation for a large fraction of our zebrafish transcriptome.

### Identification of a stringent set of zebrafish lincRNAs

**Figure 2:**
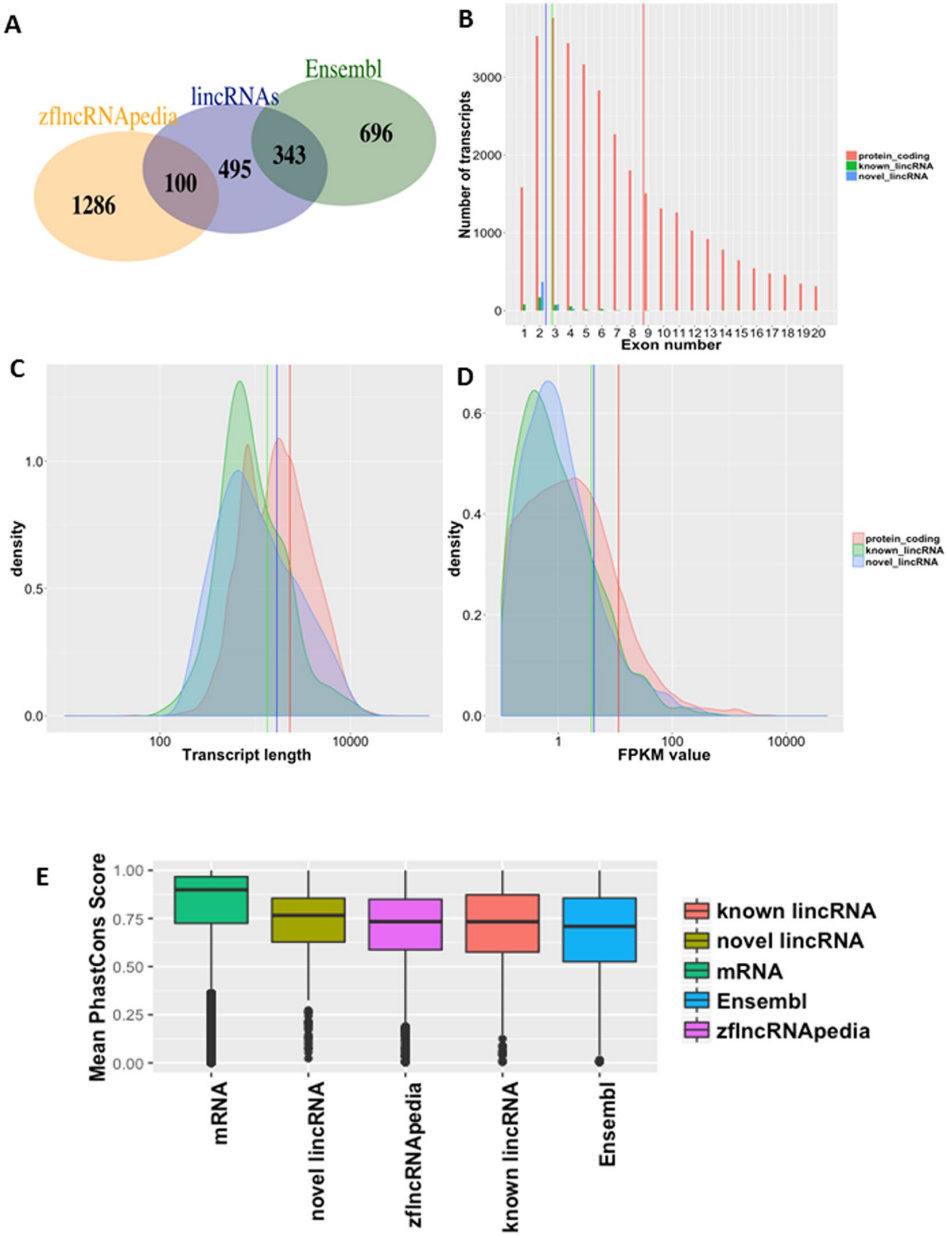
Zebrafish lincRNAs are shorter, less conserved, and expressed at lower levels than protein-coding genes. (**A**) Overlap among cardiac lincRNAs (dark blue) and other existing annotations: Ensembl (dark green), and zflncRNApedia (orange). Distribution of exon numbers (**B**), transcript length (**C**), expression level (**D**), and phastCons scores (**E**) for protein-coding genes, previously annotated lincRNAs, and novel lincRNAs in our RNA-seq dataset.

Using our transcriptome derived from zebrafish heart and muscle, we applied 6 filters in a stringent bioinformatics filtering pipeline to identify putative lincRNAs (Fig. 1A). First, we removed candidate transcripts that overlapped with known protein-coding genes in the zebrafish Ensembl annotation (Danio_rerio.Zv9.79.gtf). Second, we removed candidate transcripts with only 1 exon, a length less than 200 nt, or a fragments per kilobase of transcript per million mapped reads (FPKM) value less than 0.1. For the remaining putative noncoding transcripts, 343 were known lincRNA genes in the Ensembl annotation, and 100 were annotated by the zflncRNApedia annotation database^36^. Third, for the remaining 1,739 novel candidate transcripts, we removed transcripts with coding potential using the Cording Potential Assessing Tool (CPAT)^37^ based on the size of the open-reading frames, open-reading frame coverage, Fickett TESTCODE statistics, and hexamer bias. This filter retained 863 candidate putative noncoding transcripts. Fourth, we removed candidate transcripts that had high similarity to known proteins or protein domains in the Pfam database using the Hidden Markov Model-based sequence alignment tool (HMMER)^38^. Candidate transcripts with a significant Pfam hit (*P* < 1 × 10^−5^) were removed, which resulted in 707 candidate noncoding transcripts. Fifth, we evaluated the protein-coding potential of these 707 transcripts with the Coding Potential Calculator (CPC) algorithm based on 6 biologically meaningful sequence features^39^. This filter retained 565 putative noncoding transcripts. Sixth, we used the Phylogenetic Coding Substitution Frequency (PhyloCSF) tool to score the coding potential of transcripts using multispecies nucleotide sequence alignment^40^. We chose a PhyloCSF threshold of less than 20 because it retained most of the RefSeq ncRNAs^41^. The resulting set contained 495 novel lincRNAs. In total, we identified 938 lincRNAs with detectable expression (i.e., FPKM value >0.1), 443 of which were known lincRNAs (i.e., 343 lincRNAs from the Ensembl annotation Zv9 and 100 from the zflncRNApedia database) and 495 of which were novel lincRNAs (Fig. 2A).

**Figure 3:**
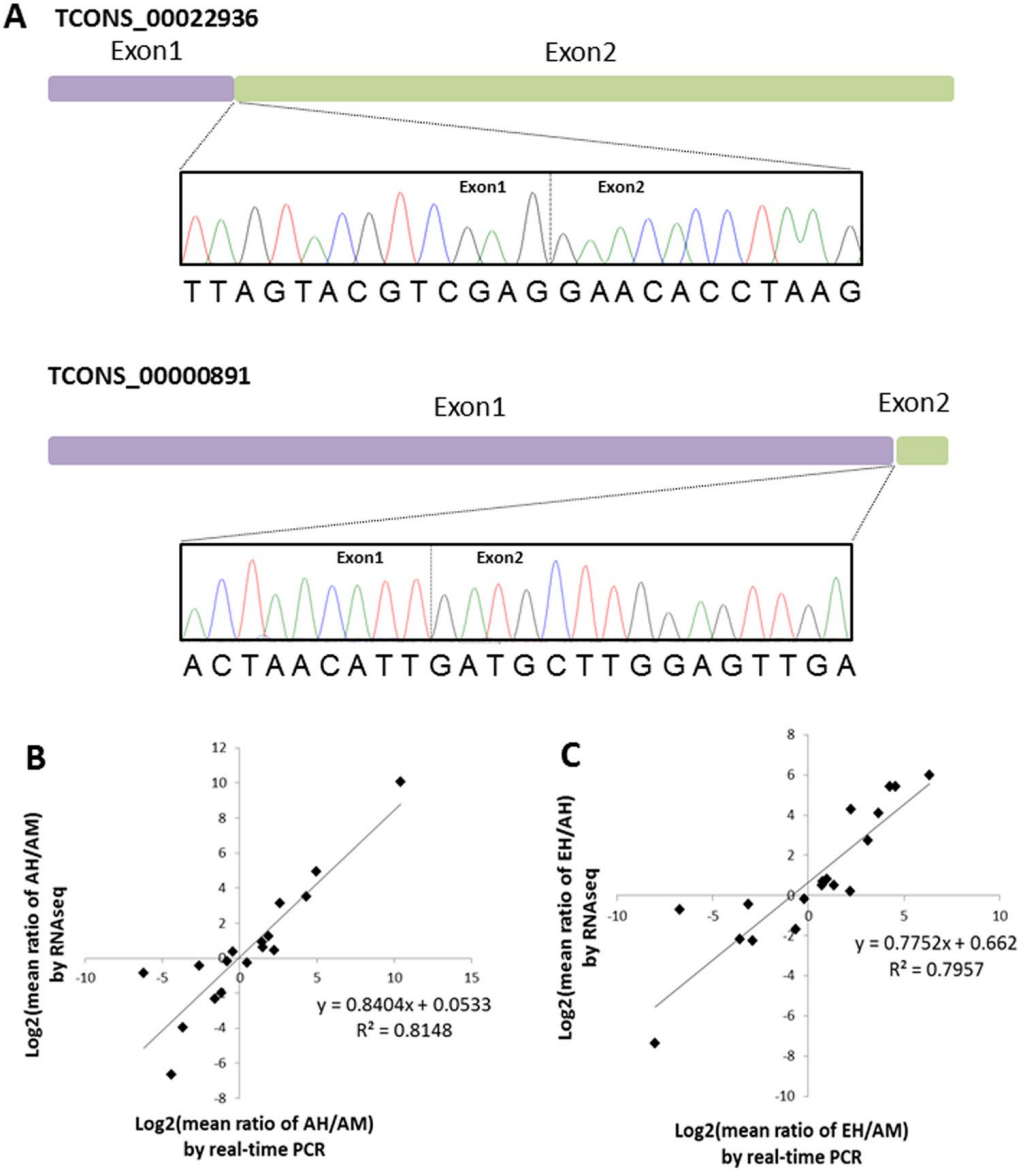
Validation of RNA-seq–predicted lincRNAs. (**A**) Sanger sequence validation of exon junction regions of 2 novel lincRNAs, TCONS_00022936 and TCONS_00000891. Sequence of TCONS_00000891 is shown in reverse complement form. (**B**) Quantitative real-time PCR validation of expressions of 17 lincRNAs between adult fish heart (AH) and adult fish muscle (AM). (**C**) Quantitative real-time PCR validation of expressions of 18 lincRNAs between embryonic fish heart (EH) and adult fish heart.

To assess the gene structure of lincRNAs reconstructed by our bioinformatics pipeline, we experimentally tested 5 randomly selected lincRNAs, including 2 novel predicted lincRNAs identified by our pipeline (TCONS_00022936 and TCONS_00000891) and 3 lincRNAs that overlap with known transcripts (TCONS_00048108, TCONS_00009015, and TCONS_00053013). We cloned all 5 lincRNAs from the zebrafish cardiac cDNA library and confirmed all exon junction regions via Sanger sequencing (Fig. 3A and Supplemental Figure 1). These data proved the fidelity of the gene structure of the lincRNA transcripts identified by our bioinformatics pipeline.

To assess the expression level of lincRNAs detected by our RNA-seq dataset, we used quantitative reverse-transcription polymerase chain reaction (qRT-PCR) with a selected set of 17 lincRNAs to compare their relative expression levels between adult heart and adult muscle. We observed a correlation coefficient of 0.81 between readouts from RNA-seq and qRT-PCR experiments (Fig. 3B). Similarly, comparing the relative expression levels of 18 lincRNAs between embryonic heart and adult heart resulted in a correlation coefficient of 0.79. These results proved the fidelity of the expression levels of the lincRNAs from our RNA-seq data set (Fig. 3C).

### Molecular and expression properties of zebrafish lincRNAs

Many genome-wide studies have demonstrated that lincRNAs are shorter, less conserved, and expressed at significantly lower levels than protein-coding genes^32, 42, 43^. Analyzing the structures, expression levels, and conservation of 938 lincRNAs in our RNA-seq dataset showed that these lincRNAs had fewer exons per transcript (2.8 exons for known lincRNAs; 2.4 exons for novel lincRNAs) than protein-coding transcripts (8.7 exons; Fig. 2B). The novel lincRNAs also exhibited a length density distribution similar to that of known lincRNAs (a mean length of 1,343 nt for known lincRNAs, and a mean length of 1,692 nt for novel lincRNAs), both of which are shorter than protein-coding transcripts (mean length, 2,324 nt) (Fig. 2C). Among the lincRNA transcripts with mean FPKM values higher than 0.1 in at least 1 of the 3 tissue types, lincRNAs were expressed at lower levels than protein-coding transcripts (on average, 8.7 FPKM for protein-coding transcripts, 3.8 FPKM for known lincRNAs, and 4.2 FPKM for novel lincRNAs; Fig. 2D). Based on the phastCons scores, the conservation level of novel lincRNAs was similar to those of known lincRNAs in the Ensembl and zflncRNApedia databases^36^, which are substantially lower than those of protein-coding transcripts (Fig. 2E).

**Figure 4:**
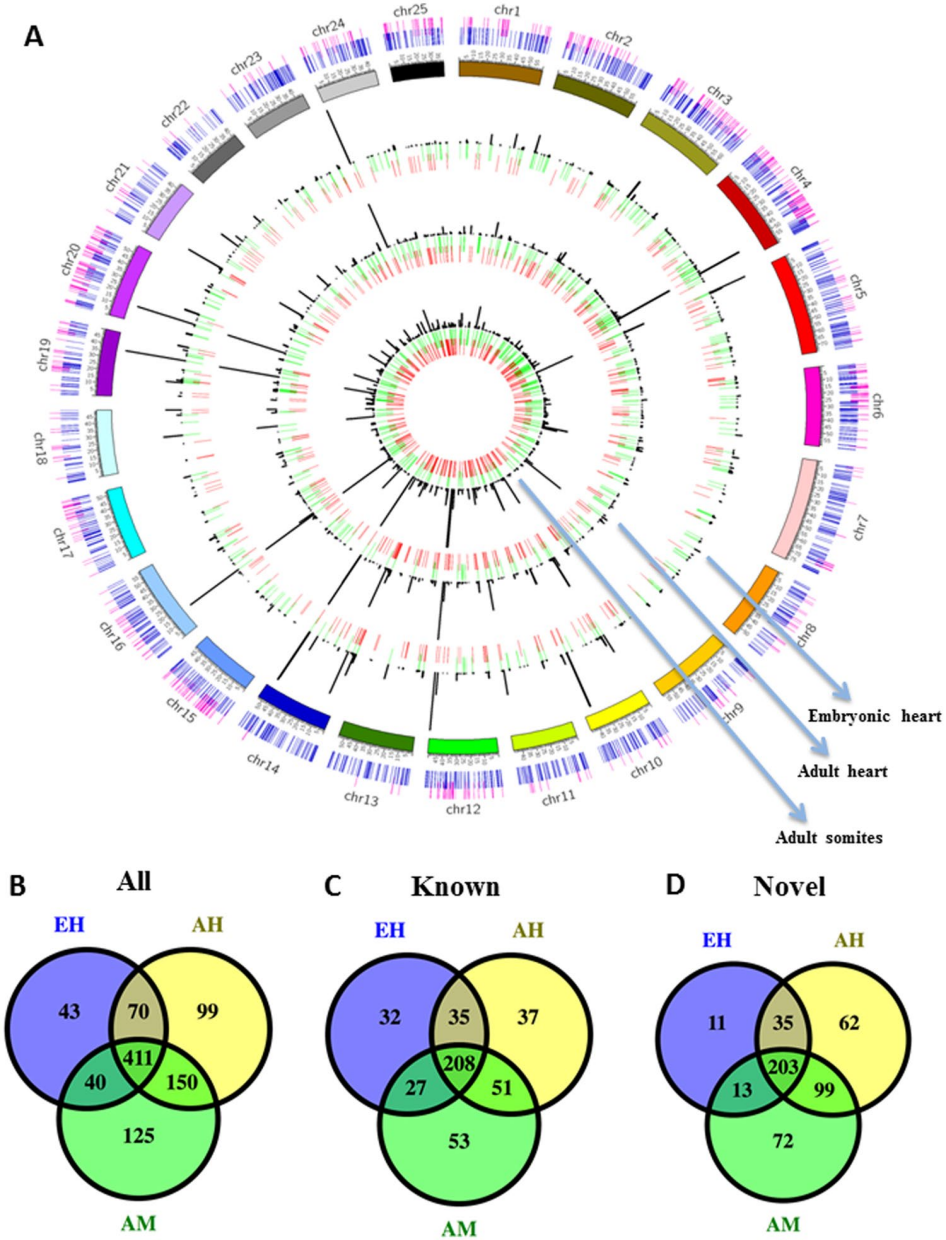
Overview of lincRNA distribution across the reference genome and different tissue types. (**A**) A ubiquitous distribution of cardiac lincRNAs in the zebrafish genome and spatiotemporal expression patterns (putative novel lincRNAs, red; annotated lincRNAs, green; the FPKM value (FPKM <50) for histogram). Also shown is the number of (**B**) all lincRNA transcripts; (**C**) known lincRNA transcripts; and (**D**) novel lincRNA transcripts expressed in 3 tissue types and their overlaps. AH, adult heart; AM, adult muscle; EH, embryonic heart.

Because lincRNAs in the human genome are ubiquitously distributed across all chromosomes^44^ but have tissue-specific expression^32^, we investigated the genomic distribution of 938 lincRNAs and their expression patterns in the 3 muscular tissue types in zebrafish. These lincRNAs were distributed across all chromosomes in the zebrafish reference genome (Fig. 4A). Overall, 601 lincRNAs were expressed in both heart and muscle tissues, and 212 lincRNAs displayed a putative enriched expression pattern in heart tissues but not muscle. Thus, we termed the 813 heart-expressed lincRNAs the *cardiac lincRNA catalog*. Among the 212 cardiac-specific lincRNAs, 99 were expressed only in the adult heart, and 43 were expressed only in the embryonic heart (Fig. 4B). Notably, 62 of the 99 adult heart-specific lincRNAs and 11 of the 43 embryonic heart-specific lincRNAs were novel (Fig. 4C and D). The remaining 125 lincRNAs showed more enriched expression in the muscle tissue instead of in the cardiac tissue (Fig. 4B). Thus, approximately 36% of the lincRNAs (212 in the heart and 125 in the muscle) showed a tissue-enriched expression pattern.

### Analysis of embryonic heart-enriched lincRNAs

**Figure 5:**
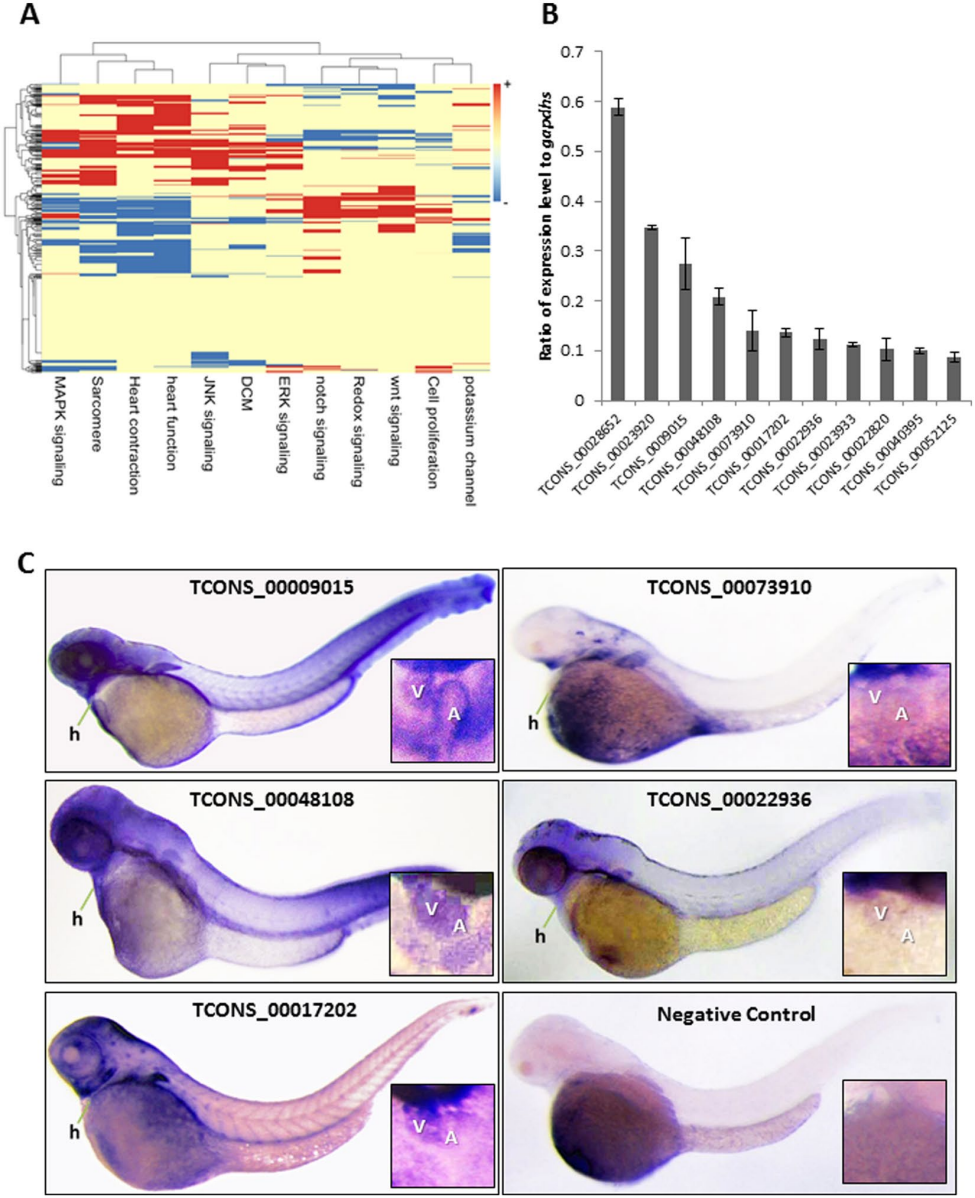
Analysis of embryonic heart–enriched lincRNAs. (**A**) Expression-based association matrix of 564 embryonic heart–enriched lincRNA transcripts (rows) and functional gene sets (columns). Red, positive correlation; blue, negative correlation; yellow, no correlation. (**B**) Expression of embryonic heart-enriched lincRNAs, as revealed by Quantitative real-time PCR. *gapdhs* (FPKM = 127) was used as reference gene. Means ± S.D. N = 3. (**C**) Validation of 5 selected lincRNAs with high embryonic cardiac expression by *in situ* hybridization at 2-days post fertilization (2 dpf), and negative control is a lincRNA candidate with low FPKM in embryonic heart. A, atrium; h, heart; V, ventricle.

Among the 813 cardiac lincRNAs, 564 were expressed in the embryonic heart (i.e., mean FPKM value >0.1); of these, 302 (53.5%) were known (i.e., annotated in Ensembl or the zflncRNApedia database), and 262 (46.5%) were putative novel lincRNAs (Supplemental Table 1). In the gene functional enrichment analysis by the Ingenuity Pathway Analysis (IPA) tool (http://www.ingenuity.com/), the neighboring protein-coding genes of embryonic heart-enriched lincRNAs were significantly enriched in development-related biological processes, such as embryonic development, organismal development, and tissue morphology (Supplemental Table 2). Since lincRNAs have a greater likelihood to be functionally associated with their nearest neighboring protein-coding genes^45, 46^, it is likely that these lincRNAs are also involved in same biological processes. Notably, 21 embryonic heart-enriched lincRNAs were bidirectional lincRNAs (Supplemental Table 3). Based on a gene set analysis (GSA) between embryonic heart-enriched lincRNAs and functional gene sets associated with heart development and disease (Supplemental Table 4), the embryonic heart-enriched lincRNAs were significantly associated with mitogen-activated protein kinase (MAPK) signaling, heart function, heart contraction, and sarcomere organization (Fig. 5A).

For the 11 lincRNAs with the highest expression levels in embryonic heart (i.e., mean FPKM value >25 in all 3 biological replicates), their expression levels were between 10% and 60% of that of *gapdhs*, a housekeeping gene with an average FPKM of 127 in embryonic heart^47^ (Fig. 5B). In whole-mount *in situ* hybridizations targeting these lincRNAs, cardiac expression was confirmed for 5 lincRNAs in both the atrium and ventricle of 2dayspostfertilization (dpf) embryos. Instead of a cardiac or muscle restricted expression pattern, these lincRNAs were also expressed in other tissues, including brain, muscle, pectoral fin, and tail (Fig. 5C). Dynamic expression was noted for some lincRNAs during embryogenesis. For example, longitudinal studies of TCONS00009015 revealed no cardiac expression at 1 dpf, weak cardiac expression at 2 dpf, and strong cardiac expression at 4 dpf (Supplemental Figure 2).

### Analysis of adult heart-enriched lincRNAs

**Figure 6:**
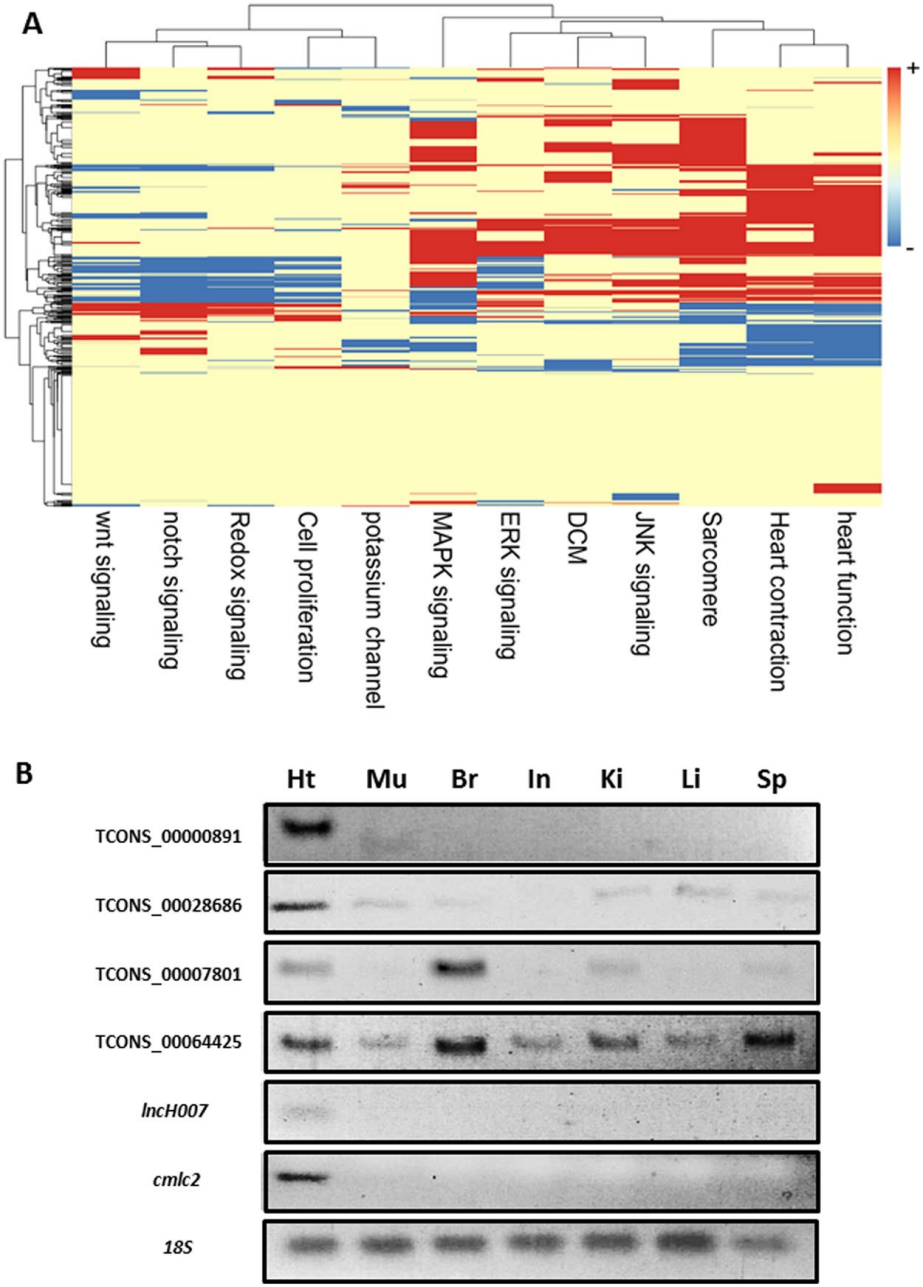
Analysis of adult heart–enriched lincRNAs. (**A**) Expression-based association matrix of 730 adult heart–enriched lincRNA transcripts (rows) and functional gene sets (columns). Red, positive correlation; blue, negative correlation; yellow, no correlation. (**B**) Tissue-specific expression pattern of adult heart-enriched lincRNAs across 7 tissues. Semi-quantitative RT-PCR of adult cardiac–enriched lincRNAs in 7 fresh tissue samples. PCR products were electrophoresed in 3% agarose gel. 18 S served as internal control and *lncH007* and *cmlc2* served as cardiac-specific positive controls. Br, brain; Ht, heart; In, intestine; Ki, kidney; Li, liver; Mu, muscle; Sp, spleen. Gel images are cropped around 100 bp-300 bp region. Product size information is listed in Supplemental Table 8.

In adult zebrafish heart, 730 lincRNAs had detectable expression levels (i.e., mean FPKM value >0.1); of these, 331 (45.3%) were known, and 399 (54.7%) were putative novel lincRNAs (Supplemental Table 1). In the gene functional enrichment analysis (i.e., IPA) of adult heart-enriched lincRNAs, the neighboring protein-coding genes of these lincRNAs were significantly enriched in basic biological processes, such as RNA postmodification and cardiovascular system development and function (Supplemental Table 2). Notably, 29 of the adult heart-enriched lincRNAs were bidirectional lincRNAs (Supplemental Table 3). Based on a GSA between adult heart-enriched lincRNAs and functional gene sets associated with heart development and disease, the adult heart-enriched lincRNAs were associated not only with MAPK signaling, heart function, heart contraction, and sarcomere organization, but also with extracellular signal-regulated kinase (ERK) signaling, c-Jun N-terminal protein kinase (JNK) signaling, and dilated cardiomyopathy (DCM) (Fig. 6A).

We selected 21 lincRNAs with the highest expression ratios between adult heart and adult muscle for downstream experimental validation. The FPKM ratios of these lincRNAs between the heart and muscle groups ranged from 1,071 to 1.3 (Supplemental Table 5). We determined the expression levels of these lincRNAs in 7 adult zebrafish tissues—heart, muscle, brain, intestine, kidney, liver, and spleen—using semi-quantitative PCR. Tissue-specific expression patterns were found for several candidates. TCONS_00000891 was only expressed in heart, similar to the cardiac-restricted expression patterns of *lncH007*, a known cardiac lincRNA, and *regulatory myosin light chain* (*cmlc2*), a known cardiac gene^48^ (Fig. 6B). TCONS_00028686 was expressed predominantly in heart rather than the other tissue types; TCONS_00040108 was expressed in heart, brain, and kidney; TCONS_00022820 was expressed in heart and spleen; TCONS_00007801 was expressed in heart and brain; and TCONS_00064425 was expressed in heart, brain, kidney, and spleen (Fig. 6B and Supplemental Figure 3).

### Analysis of fetal lincRNAs

**Figure 7:**
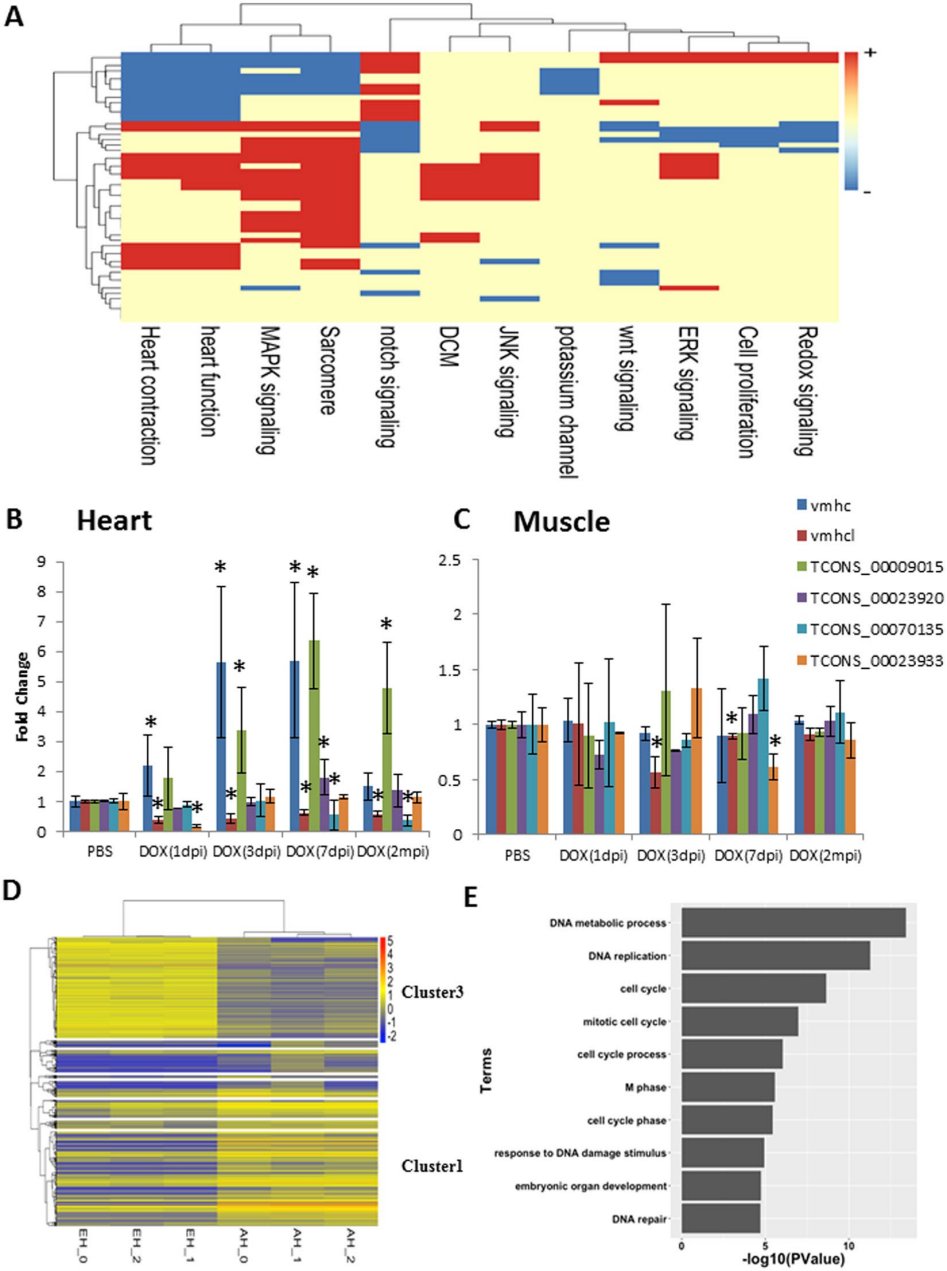
Analysis of fetal lincRNAs. (**A**) Expression-based association matrix of 51 fetal lincRNA transcripts (rows) and functional gene sets (columns). Red, positive correlation; blue, negative correlation; yellow, no correlation. Expression of fetal lincRNAs upon DOX stress in adult heart and muscle. Quantification of expression level change of lincRNAs upon DOX stress in the heart (**B**) and in the muscle (**C**). Means ± S.D. N = 5. *P < 0.05 compared to control (PBS) group. Ratio of *vmhc* to *vmhcl* served as cardiac remodel marker. (**D**) K-means clustering for all differentially expressed transcripts including protein-coding transcripts and lincRNAs. (**E**) Gene functional enrichment analysis of protein-coding genes in Cluster 3 suggested that fetal lincRNAs (all grouped into Cluster 3) were significantly enriched in several biological processes including DNA metabolic processes, DNA replication, and cell cycle (FDR <0.01).

In the cardiac transcriptome, a subgroup of developmentally active genes, dubbed the *fetal gene program*, is particularly interesting. These genes are characterized by differential expression between embryonic and adult hearts. It is hypothesized that many of these quiescent genes have important roles during cardiac diseases, especially those that are reactivated during pathogenesis upon stress. Similar to the fetal gene program, the fetal lincRNA program has been explored recently in mouse models^49^. Using our definition of the fetal lincRNA program in a zebrafish heart (i.e., the lincRNA expression level is at least 4-fold different between embryonic heart and adult heart^50^), we identified 51 fetal lincRNAs (Supplemental Table 6). To investigate biological processes associated with these putative fetal lincRNAs, we did a GSA between fetal lincRNAs and all 8047 biological processes in the ZebrafishMine database (http://www.zebrafishmine.org/). However, only few biological processes showed correlation relationship with fetal lincRNAs (Supplemental Figure 4). To further investigate whether these putative fetal lincRNAs are associated with cardiac-remodeling biological processes, we manually collected a list of biological processes associated with heart development and diseases (Supplemental Table 4) and performed a GSA between the expression level of fetal lincRNAs and that of genes in these biological processes. The expression level of most fetal lincRNAs was positively or negatively correlated with our manually curated heart-related biological processes, including MAPK signaling, heart function, heart contraction, potassium channel, sarcomere functional gene sets, and the notch signaling gene set (Fig. 7A).

To identify fetal lincRNAs that can be reactivated under cardiac stress, we investigated the expression change of 15 fetal lincRNAs with higher expression levels in embryonic heart in an established DOX-induced cardiomyopathy (DIC) model in zebrafish^51^. Four fetal lincRNAs exhibit changes of expression upon DOX stress, while other 11 fetal lincRNAs remain unchanged. TCONS_00009015 was activated by at least 4-fold upon DOX stress at 1 day post injection (dpi), which remained highly expressed until 2 months post injection. TCONS_00023920 was only induced temporarily at 1 week after the DOX injection. By contrast, expression of TCONS_00070135 started to decrease at 7 dpi of DIC, and expression of TCONS_00023933 was reduced at 3 dpi and recovered at later time (Fig. 7B). We did not observe similar inductions in the muscle, which suggests that the transcriptional activation is specific to cardiac stress induced by DOX (Fig. 7C).

Unsupervised K-means clustering for all differentially expressed transcripts (i.e., protein-coding transcripts and lincRNAs) showed that all 16 fetal lincRNAs that were highly expressed in embryonic heart were grouped into Cluster 3, whereas 25 of the 35 (71%) lincRNAs that were highly expressed in adult heart were in Cluster 1 (Fig. 7D). Gene functional enrichment analysis of the protein-coding genes in Cluster 3 by the Database for Annotation, Visualization and Integrated Discovery (DAVID) v6.8^52^ suggested that the transcripts in Cluster 3 were significantly enriched in several biological processes, including DNA metabolic processes, DNA replication, and cell cycle (false discovery rate [FDR] <0.01; Fig. 7E). In contrast, as shown by IPA network enrichment analysis, the transcripts in Cluster 1 are significantly involved in various biological functions, including cardiac hypertrophy, cardiovascular disease, and developmental disorders, which suggest that the lincRNAs highly expressed in adult heart are involved in similar biological processes (Supplemental Table 2).

## Discussions

To systematically identify and characterize the lincRNAs associated with heart development and cardiac diseases, we generated a reference catalog of 813 cardiac lincRNAs in the zebrafish model by integrating RNA-seq data from 3 tissues with publicly available transcript annotations. In all, 423 (52%) of the transcripts in the cardiac catalog are novel lincRNAs identified using our RNA-seq dataset. Characterizing these lincRNAs via structural, expression, and evolutionary analyses indicated that cardiac lincRNAs share similar features with other lincRNA annotations in the public domain. Our cardiac lincRNA catalog will shed new light on future experimental and computational studies investigating lincRNA function in heart-associated biological processes and disease mechanisms.

Similar genome-wide lncRNA studies in the heart have been conducted recently in human, mouse, and zebrafish. Using RNA-seq, Chunjiang *et al*. identified the contrasting coordination of *cis*- and *trans*- molecular regulation through lncRNAs expressed in human fetal and adult hearts^50^. Scot *et al*. used comprehensive quantitative RNA-seq data from mouse hearts, livers, and skin cells to identify both cardiac-expressed and cardiac-specific lncRNAs^53^. Kriti *et al*. combined RNA-seq with computational analysis to identify tissue-restricted lncRNA signatures in the heart, liver, muscle, brain, and blood of adult zebrafish^48^. Compared with these studies, our study on zebrafish cardiac lincRNAs is unique in several ways. First, we focused on lincRNAs because their specific genomic locations facilitate downstream experimental manipulation and computational analysis. Our independent experimental validation, which included Sanger sequencing, qRT-PCR, *in situ* hybridization, and DOX stress experiments, suggested that our cardiac lincRNA catalog is a high-quality lincRNA set that will facilitate experimental studies and further functional classification of these lincRNAs, some of which might serve as key regulators in biological processes involved in cardiac diseases. Second, we used more comprehensive reference annotation resources, including RefSeq, Ensembl, and zflncRNApedia, to identify the known lincRNAs expressed in our dataset, which helped improve the accuracy of our lincRNA identification. Third, we developed an integrative yet conservative bioinformatics filtering workflow that enabled us to identify new cardiac lincRNAs by generating a high-confidence cardiac lincRNA catalog. Cardiac lncRNA studies in mouse^53^ and human^50^ have focused only on known lncRNAs, which constitute only a small subset of lncRNAs^14^. Kriti *et al*. identified both known and novel lncRNAs from 5 different tissues of adult zebrafish. In contrast, our study used a more comprehensive and stringent bioinformatics filtering strategy to identify putative cardiac lincRNAs, several of which were further independently confirmed by biological experiments. Finally, we identified several novel lincRNAs expressed specifically in heart tissues. Among the 813 cardiac lincRNAs, 564 were expressed in embryonic heart, and 730 were expressed in adult heart. We also identified 16 fetal lincRNAs with much higher expression levels in embryonic heart than adult heart and 35 fetal lincRNAs with much higher expression levels in adult heart than embryonic heart. Specifically, TCONS_00000891 was expressed only in heart but not in other organs, whereas TCONS_00009015 was a unique fetal lincRNA that might play important biological roles after cardiac stresses, similar to known cardiac-remodeling hallmarks, such as the ratio between *ventricular myosin heavy chain* (*vmhc*) and *ventricular myosin heavy chain-like* (*vmhcl*), functional zebrafish orthologues for human genes *myosin heavy chain 6* (*MYH6*) and *MYH7* (Fig. 7)^35^. In contrast with *mhrt*, a cardiac protective ncRNA that is also an antisense transcript of *MYH7* ^54^, the location of TCONS_000009015 does note overlap with the loci of either *vmhc* or *vmhcl* on the zebrafish genome, indicating that it might be involved in cardiac remodeling process via a different mechanism. Further investigation of these cardiac lincRNAs is warranted and might lead to the discovery of novel disease biomarkers or targets for therapeutic intervention.

Mechanistically, lincRNAs can regulate gene expression in both *cis* and *trans* manners^32^. For instance, lincRNAs have been shown to perform *cis*-acting regulation of target genes located at or near the same genomic locus in human heart^50^. Mammalian lincRNAs are enriched near protein-coding genes encoding for transcription factors and genes involved in nervous system development^55–57^. The neighboring genes of zebrafish lincRNAs have also been found to be enriched in transcriptional processes^43^. In the present study, the neighboring genes of zebrafish lincRNAs from all 3 tissues were enriched in transcription regulatory processes: “transcription regulator activity” (GO:0030528) and “transcription factor activity” (GO:0003700). We found that the neighboring genes of cardiac-specific lincRNAs are enriched in muscle component categories, such as troponin complex and striated muscle thin filament. Among these cardiac-specific lincRNAs, 9 are bidirectional lincRNAs located within 1,000 bases of a neighboring protein-coding gene on the opposite strand. LincRNAs of this specific subclass have been shown to serve as bidirectional promoters for neighboring protein-coding genes^58, 59^. Within this subclass, TCONS_00016569 is a novel bidirectional lincRNA, and its counterpart protein-coding gene, *cacna1g*, is involved in calcium channel activity based on GO annotation. TCONS_00014804 is a known lincRNA (ENSDART00000152911) with an unknown function, and its counterpart protein-coding gene, *Zcchc4*, is associated with methyltransferase activity based on GO annotation. Thus, our cardiac lincRNA catalog should facilitate the generation of new hypotheses regarding the potential roles of these lincRNAs in cardiac processes and disease mechanisms.

In addition to *cis* effects, lincRNAs have also been shown to primarily regulate gene expression in *trans* by loss-of-function studies of hundreds of lincRNAs expressed in mouse embryonic stem cells^19^. Based on the “guilt-by-association” rule^56, 60^, we observed that some cardiac lincRNAs were coexpressed with several heart-associated functional gene sets (Supplemental Table 4), which indicates that cardiac-specific lincRNAs are associated with heart development and regulation and may be potential regulators in heart diseases. Hierarchical clustering analysis of differentially expressed transcripts (including both lincRNAs and protein-coding transcripts) showed that most fetal lincRNAs were grouped into Clusters 1 and 3 with protein-coding transcripts that are significantly enriched in several biological processes, including DNA metabolic processes, DNA replication, and cell cycle (Fig. 7D and E). Thus, these cardiac lincRNAs may exert their functions by participating in biological processes similar to those of their coexpressed protein-coding partners. For instance, TCONS_00028686 in Cluster 1 had high expression in adult heart, and its genomic location overlaps with that of *lncH_009*, a known lincRNA with expression only in the adult heart in zebrafish^48^. Although the detailed function of TCONS_00028686 is not known, it shares a similar expression pattern with homeobox-containing genes, which implies that it could be involved in similar biological processes.

Despite their evolutionary distance, orthologues for zebrafish lincRNAs can be found in humans and mice^30^. Using multispecies genome alignments, we identified 72 human orthologous regions for 65 zebrafish lincRNAs and 67 mouse orthologous regions for 57 zebrafish lincRNAs (Supplemental Table 7). Among these orthologous regions, 14 human orthologous regions have annotations in human GENCODE (Release 19), and 11 mouse orthologous regions have annotations in mouse GENCODE (Release M1). Notably, the lincRNA TCONS_00071932 mapped to the human lincRNA LINC00672. Low-level conservation across different organisms may be attributable to the importance of transcription from a specific genomic location, reduced selective pressure on the primary sequence of ncRNAs, or the rapid evolution of new functions. Our analysis was limited by the available transcript annotations in other species, which will be improved as more transcriptomes are sequenced in other organisms in the future.

In summary, our study provides a high-confidence catalog of cardiac lincRNAs in the zebrafish model, laying a solid foundation for future studies. First, changes in these cardiac lincRNAs during the pathogenesis of different cardiac disease models can be examined and reveal candidate lincRNAs that play vital roles during pathogenesis. Second, the specific functions of promising lincRNAs must be elucidated via loss-of-function and gain-of-function studies. We anticipate that these future studies will significantly improve our understanding of the biological functions of cardiac lincRNAs.

## Materials and Methods

### Zebrafish husbandry

Wild-type WIK zebrafish (Danio rerio) were maintained under a 14-hour light/10-hour dark cycle at 28.5 °C. This study was approved by the Mayo Clinic Institutional Animal Care and Use Committee (A17515). All study procedures were performed in accordance with the Guide for the Care and Use of Laboratory Animals^61^.

### RNA-seq dataset

We used the RNA sequencing (RNA-seq) dataset from our previous study^35^ for zebrafish cardiac transcriptome reconstruction. Nine biological samples from 3 groups (3 samples of adult heart tissue, 3 samples of adult muscle tissue, and 3 samples of embryonic heart tissue) were sequenced using the HiSeq 2000 platform (Illumina, San Diego, CA, USA) with a 50-bp pair-end sequencing protocol in the Mayo Clinic DNA Sequencing Core Facility.

### Transcriptome assembly

Raw RNA-seq reads for each sample were aligned by TopHat (Version 2.0.12) to the zebrafish genome assembly (Zv9) using the Ensembl annotation Zv9 (Danio_rerio.Zv9.79.gtf)^62^. Each transcriptome was assembled by Cufflinks (Version 2.2.1)^63^. Transcripts were considered expressed if they had an FPKM value greater than 0.1 in at least 2 of 3 samples in the same group according to the Cuffnorm script from Cufflinks. Transcripts were considered to be differentially expressed across different groups if they exhibited a greater than 2-fold change and an FDR of less than 0.05 according to the Cuffdiff script from Cufflinks.

### Bioinformatics filtering workflow to identify high-confidence lincRNAs

In the reconstructed transcriptome, transcripts designated in the ‘u’ category by Cufflink (i.e., unknown intergenic transcripts) were obtained using the Cuffcompare script with the Ensembl annotation file (Danio_rerio.Zv9.79.gtf). The annotated lincRNAs in Ensembl were also obtained in this step. The annotated lincRNAs in the zflncRNApedia database were obtained using the Cuffcompare script with the zflncRNApedia annotation^36^. The remaining novel candidate transcripts were then filtered in 5 steps: 1) FPKM value greater than 0.1; 2) sequence length longer than 200 nt; 3) exon number greater than 1; 4) evaluation of coding potential using CPAT, CPC, and PhyloCSF; and 5) filtration of candidates with known protein domains using Pfam. An overview of this process is shown in Fig. 1A.

### Conservation analysis

To examine the sequence conservation of lincRNAs, we used the phastCons scores derived from the University of California, Santa Cruz (UCSC) 8-way vertebrate genome alignment seeded with the zebrafish reference genome (Zv9)^64^. The phastCons scores for the whole genome were downloaded from the UCSC database (http://hgdownload.cse.ucsc.edu/goldenPath/danRer7/phastCons8way). The phastCons score of each exon was defined as the average phastCons score of each nucleotide of that exon. Exons with no phastCons scores were ignored.

### Identification of orthologous loci across zebrafish, mouse, and human

The lincRNAs in zebrafish were mapped to the human reference genome hg19 and the mouse reference genome mm9 through the UCSC database^65^ using the bedToPsl, pslMap, pslToBed, and pslCDnaFilter scripts. This process was guided by the chain datasets (danRer7.hg19.all.chain and danRer7.mm9.all.chain) from the UCSC database (http://hgdownload.cse.ucsc.edu/downloads.html).

### Clustering analysis

Unsupervised hierarchical clustering was performed with Pearson correlation and complete linkage options using the pheatmap R package (https://github.com/raivokolde/pheatmap). To infer potential functions of differentially expressed lincRNAs across tissue types, K-means clustering analysis was applied to all differentially expressed transcripts, including lincRNAs and protein-coding transcripts. The optimal k value was determined based on the average silhouette width using the fpc R package^66^.

## GSA

GSA was performed to infer the potential functions of cardiac lincRNAs. The Pearson correlation coefficient between each lincRNA and each protein-coding gene in each functional gene set was calculated by the GSA R package^67^, as described previously^56, 68^. The functional gene sets included heart-associated gene sets annotated in the Zebrafishmine database (www.zebrafishmine.org; Supplemental Table 4) and our previous DCM gene set^35^. The GSA scores of all functional gene sets were transformed to a ternary scale (−1, 0, 1): Gene sets with FDR >0.5 were assigned a value of 0, gene sets with FDR <0.5 and GSA score >0 were assigned a value of 1, and gene sets with FDR <0.5 and GSA score <0 were assigned a value of −1. The score matrix was clustered and visualized with Pearson correlation distance metric and complete linkage options using the pheatmap R package.

### Functional enrichment analysis

Functional enrichment analysis was performed using the Database for Annotation, Visualization and Integrated Discovery (DAVID) tool^52^.

### Pathway and network enrichment analysis

The gene lists of interest were annotated by IPA (QIAGEN) (http://www.ingenuity.com/). We queried IPA with the gene list of interest to map and generate putative biological processes/functions, networks, and pathways based on the manually curated knowledge database of molecular interactions extracted from the public literature. The enriched pathways and gene networks were generated using both direct and indirect relationships/connectivity. These pathways and networks were ranked by their enrichment score, which measures the probability that the genes were included in a network by chance.

### qRT-PCR

RNA was extracted and purified from freshly dissected tissues using TRIzol Reagent (Thermo Fisher Scientific) following the manufacturer’s protocol. In total, 500 ng of RNA from each tissue was used to prepare cDNA using the SuperScript III First-Strand Synthesis System (Thermo Fisher Scientific). A qRT-PCR was conducted using a LightCycler 480 QPCR apparatus in 96-well QPCR plates (Roche Life Science). The expression levels of the lincRNAs of interest were calculated by the −∆∆Ct (cycle threshold) method using glyceraldehyde-3-phosphate dehydrogenase (gapdh) as an internal reference. For each tissue type, at least 3 biological replicates were measured and analyzed. The primers for the lincRNAs of interest are listed in Supplemental Table 8.

### *In situ* hybridization

*In situ* hybridization riboprobes were generated from 500- to 1,000-bp PCR products using T7-tagged primer pairs. Fish embryos were collected at 1, 2, and 4 dpf. Whole-mount *in situ* hybridization was conducted as described previously^69^. Images were recorded using an Axioplan 2 microscope (Zeiss) or a Leica III dissecting microscope equipped with a Nikon digital camera.

### DOX stress experiment

Fish were exposed to DOX stress as described previously^70^. Adult WIK zebrafish were injected with DOX (Sigma) at a dose of 20 mg/g. More than 5 fish were included in each experimental group.

### Data access

All of the raw RNA-seq data files are available from the Gene Expression Omnibus (Accession #GSE85416).

## Electronic supplementary material

Supplementary Figures

Supplemental Table 1

Supplemental Table 2

Supplemental Table 3

Supplemental Table 4

Supplemental Table 5

Supplemental Table 6

Supplemental Table 7

Supplemental Table 8
