# Supplementary material for: Systematic identification and characterization of cardiac long intergenic noncoding RNAs in zebrafish
Source: Sci Rep. 2017 Apr 28;7:1250. doi: 10.1038/s41598-017-00823-3 (PMC5430783; doi:10.1038/s41598-017-00823-3)
Supplement: Supplementary file 1 — Supplementary Figures [file 41598_2017_823_MOESM1_ESM.pdf]

Systematic identification and characterization of cardiac long intergenic noncoding RNAs in zebrafish

Lei Wang, Xiao Ma, Xiaolei Xu, and Yuji Zhang

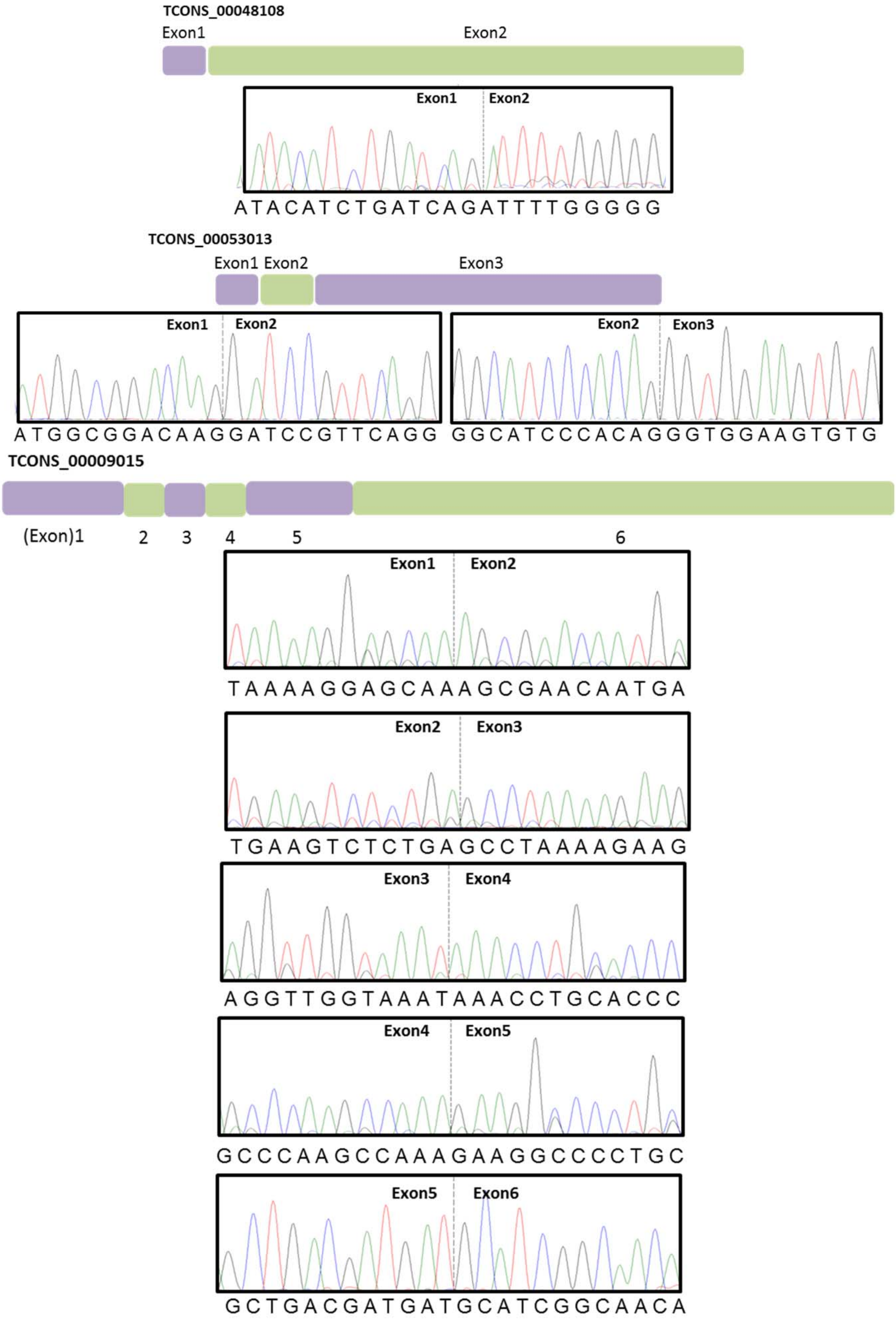

Supplemental Figure 1. Validation of three known predictive lincRNAs using Sanger sequencing approach.

TCONS_00009015

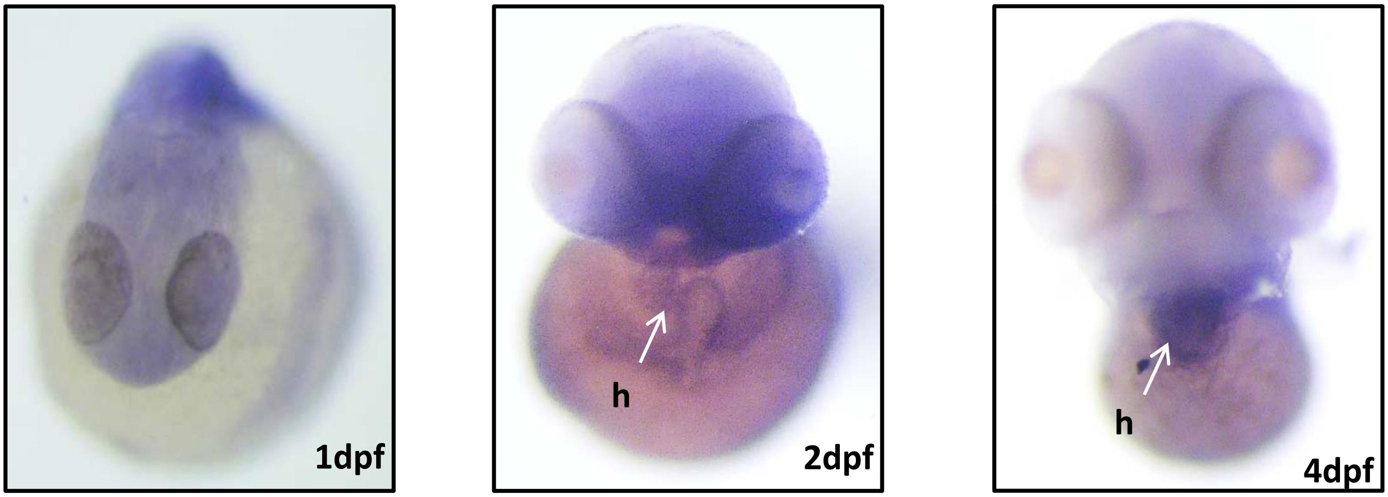

Supplemental Figure 2. Dynamics of cardiac lincRNA expression during embryogenesis.

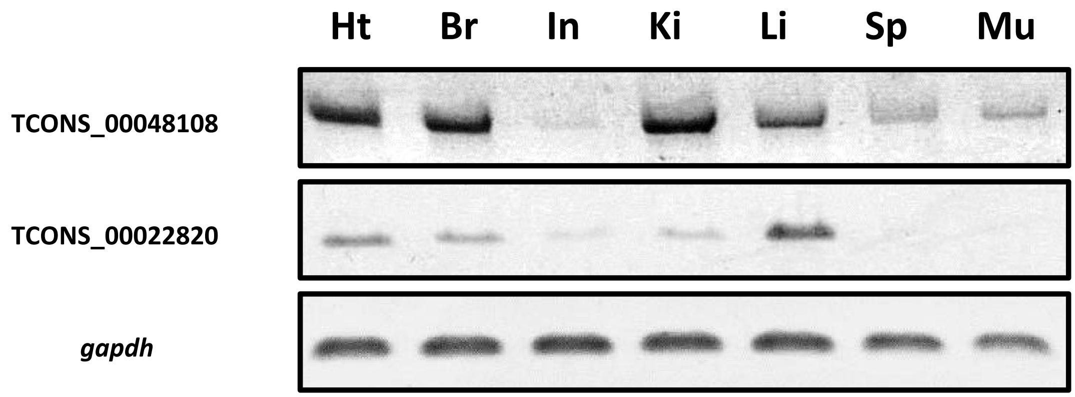

Supplemental Figure 3. Expression pattern of additional adult-enriched lincRNAs across 7 tissues. Br, brain; Ht, heart; In, intestine; Ki, kidney; Li, liver; Mu, muscle; Sp, spleen.

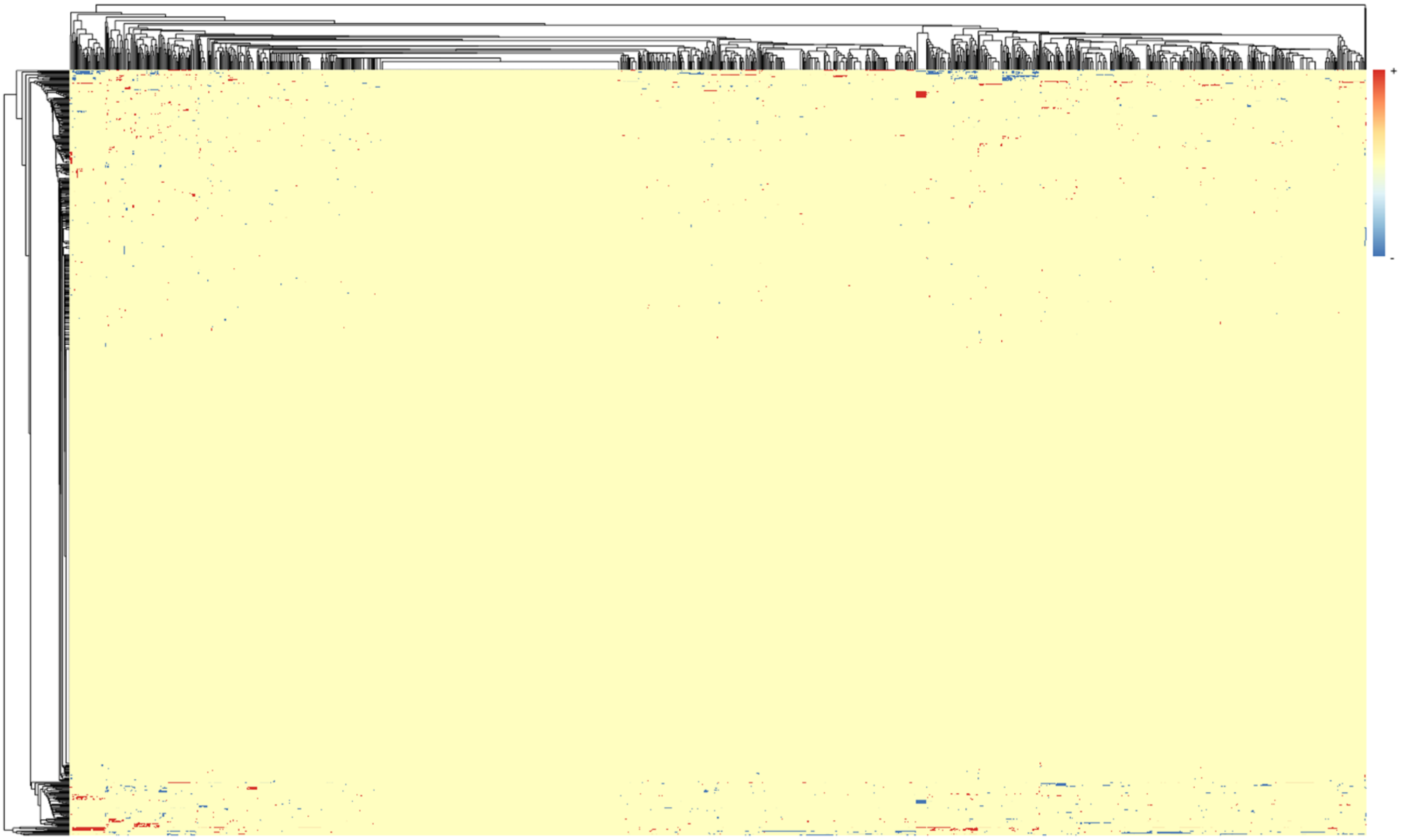

Supplemental Figure 4. Expression-based association matrix of 51 fetal lincRNA transcripts (rows) and all 8047 functional gene sets (columns). Red, positive correlation; blue, negative correlation; yellow, no correlation.
