# Supplementary material for: Systematic identification and characterization of cardiac long intergenic noncoding RNAs in zebrafish
Source: Sci Rep. 2017 Apr 28;7:1250. doi: 10.1038/s41598-017-00823-3 (PMC5430783; doi:10.1038/s41598-017-00823-3)
Supplement: Supplementary file 5 — Supplemental Table 4 [file 41598_2017_823_MOESM5_ESM.doc]

| Gene set | Annotation Source | Gene list |
| --- | --- | --- |
| DCM | DCM genes | actc1a actc1b ankrd1a ankrd1b cryaba cryabb desma desmb DSP dspa fhl2a fhl2b ldb3a ldb3b myh10 myh6 myh7ba myh7bb pdlim3a pdlim3b SCN11A scn12aa syne1a syne1b syne2a syne2b tmpoa tmpob tnnc1a tnnc1b tnnc2 tnni1a tnni1al tnni1b tnni1c tnni1d tnni2a.1 tnni2a.2 tnni2a.3 tnni2a.4 tnni2b.1 tnni2b.2 tnnt2a tnnt2c tnnt2d tnnt2e tnnt3b ttna ttnb vmhc vmhcl |
| Calcineurin_NFAT | GO | rcan1a rcan1b rcan2 rcan3 |
| ERK signaling | GO | ccl19a.1 ccl19a.2 ccl19b ccl20a.3 ccl20b ccl25b ccl32a. ccl33.3 ccl34a.4 ccl34b.1 ccl34b.3 ccl34b.4 ccl34b. ccl34b.9 ccl35.2 ccl38.1 ccl38a.3 ccl38a.4 ccl38a.5 ccl39a.10 ccl44 cds1 cds2 cetn4 cxcl32b.1 dusp3a dusp3b dusp6 fam83d garem ndrg4 nf1a nf1b pdgfaa pdgfab pdgfba pdgfbb si:dkey-25o1.7 spry1 spry2 spry4 tnip1 xcl32a.1 |
| JNK signaling | GO | aida ankrd6a ankrd6b axin1 csrp1a dusp22a dusp22b dusp3a dusp3b edar magi3a map3k12 mapk8b mapk8ip3 pigp sh3rf1 si:ch211-45c16.2 tnfrsf19 traf4a traf4b |
| MAPK signaling | GO | acsl4a admp bmp10 bmp15 bmp2a bmp2b bmp3 bmp4 bmp5 bmp6 bmp7a bmp7b bmp8a c18h3orf33 cd40 cnn2 fas gdf10a gdf10b gdf11 gdf2 gdf3 gdf5 gdf6b gdf9 gsdf hdr il11a il11b il17rd inha inhbaa inhbab inhbb lamtor1 lepa lepb lft1 lft2 mapk14a mapk14b mapkapk2a mapkapk2b mapkapk3 mstna mstnb ndr1 ndr2 ngfrb npffr2a npffr2b nradd paqr7b prok1 prok2 relt shc2 si:ch211-261n11.7 si:dkey-23c22.5 si:dkey-23c22.7 si:dkey-23c22.9 smpd2a spaw tgfa tgfb1a tgfb1b tgfb2 tgfb2l tgfb3 tnfrsf11a tnfrsf18 tnfrsf1a tnfrsf1b tnfrsf9a tnfrsf9b tnfrsfa zgc:153631 zgc:153759 |
| Redox signaling | GO | aifm1 aifm4 aifm5 dldh dnajc10 dnajc16l erp44 gclc glrx glrx2 glrx3 glrx5 gsr ndor1 nme8 p4hb pdia2 pdia3 pdia4 pdia5 pdia6 prdx1 prdx2 prdx3 prdx4 prdx6 ptgesl qsox1 rrm1 rrm2 rrm2b sh3bgrl3 si:ch1073-179p4.3 si:ch211-175m2.5 srxn1 tmx1 tmx2a tmx2b tmx3 tmx4 txn txn2 txndc11 txndc12 txndc15 txndc16 txndc5 txndc9 txnl1 txnrd1 txnrd2 zgc:100906 zgc:136472 zgc:152808 zgc:152951 zgc:153284 zgc:56493 zgc:77086 |
| apoptosis | GO | casp3a casp3b casp7 cflara cflarb dffa ift88 pak2a pak2b sirt2 |
| Cell proliferation | GO | abl1 abl2 adora2aa ago3b alms1 amotl2a apc apc2 arhgef7 atp6ap1b atp6v1f barx1 bbs1 bbs4 bcl6a bcl6ab blk bmp4 bricd5 btc btg4 btk cav1 cd40 cdh2 cdk6 cdkn1ba cdkn1bb cdx1b cnot7 cntf csf3a csf3b csk cx43 cxcl11.5 cxcl11. cxcl11.7 cxcl11.8 cxcl12a cxcl18b cxcl20 dab2ipa dab2ipb dia1a dia1b dlec1 dlg1 dlg1l dll4 dner dusp22a dusp22b e2f7 eapp ecrg4a ehf eif2ak2 emp2 etv5a fam83d fas fbn2b fer fes fgf16 fgf19 fgf1a fgf22 fgfr1a fgfr1b fgfr2 fgfr3 fgfr4 fntb fyna fynb fynrk garem gas2l3 gata3 gdf6a gle1 gli1 gli2b gnl3 gper1 hbegfa hbegfb hck hdac1 hdr hgfa hoxb8a hspg2 htr1aa htr1ab ifnphi1 ifnphi2 ifnphi3 ifnphi4 igf1 igfbp1a igfbp2a igfbp2b il11a il11b il12a il15 il15l il34 il4 ing1 ing2 ing4 ing5a ing5b irf1b irf2 itga2b itgav itgb8 itk jag2b jak1 jak2a jak2b jak3 jun junba junbb jund kctd13 klf4 lck lef1 lhx1a lin28a lin28b llgl2 loxl2a loxl2b lrrc14b lyn m17 matk melk men1 mfn2 mib1 mif mir221 mir25 mir30a mob1a myl7 naa35 nat15 ndrg1a ndrg1b ndrg4 nf1a nf1b ngfra ngfrb nkx2.5 nkx2.7 nppa nppb npr1a npr2 npr3 nr2f1a nradd nrarpa nrarpb pax6a pdf pdgfaa pdgfab pdgfba pdgfbb pdgfc pes pku300 pkz plcd3b polr3g ppdpfa ppp1r9ba ppp1r9bb prdm16 prok1 prok2 ptena ptenb ptk2aa ptk2ab ptk2ba ptk2bb ptk6b ptmab puraa purba purbb rb1 rc3h1a rc3h1b relt rhbdf1a rnu12 romo1 s100a10 s100a11 sav1 scrib scrt2 sema3d sgk1 sgk2a sgk2b sgk3 sh3bp4a shha si:ch211-112c15.8 si:ch211-167j9.5 si:ch211-195b13.1 si:ch211-261n11.7 si:ch211-261n11.8 si:ch73-206d17.1 si:ch73-340m8.2 si:ch73-49h18.1 si:dkey-23c22.5 si:dkey-23c22.6 si:dkey-23c22.7 si:dkey-23c22.9 si:dkey-260g12.1 si:dkey-97a13.12 sirt2 six1a six1b six6b smarca4a smarcb1a smyd2a smyd2b snapc4 sox2 spry1 spry2 src srrt sstr1a sstr5 stat3 styk1 syk tacc1 tacc3 tbx2b tcf7l1a tcf7l1b tec tes tfap2a tfap2b tfap2c tfap2d tfap2e tgfa tgfb1a tgfb1b tgfb2 tgfb2l tgfb3 tgfbi tgif1 thpo tipin tmem127 tnc tnfrsf11a tnfrsf18 tnfrsf1a tnfrsf1b tnfrsf9a tnfrsf9b tnfrsfa tnk1 tnk2a tnk2b tob1a tob1b tp53inp1 trim71 trpm7 tyk2 usp13 uspl1 vegfaa vegfc wdr12 wt1a yes1 yrk zap70 zgc:153631 zgc:153759 zgc:153924 zgc:158862 zgc:162255 zic1 zic2a zic4 zic5 |
| notch signaling | GO | aak1b adam10a adam10b aph1b arl6 asb11 bap1 bbs1 bbs4 cad chac1 chsy1 crb2a dab2 dbl dla dlb dlc dld dlk2 dll4 egfl6 epb41l5 erh fbxw7 fezf2 foxc1a gmds her12 her15.1 her4.1 her5 her6 hey1 hey2 heyl jag1a jag1b jag2b lfng llgl1 llgl2 maml1 maml3 mib1 nlk1 notch1a notch1b notch2 notch3 notchl nrarpa nrarpb numbl otud7b pappa2 poglut1 prss23 psen1 psen2 psenen ptbp1a rbpja rfng rras si:ch73-139j3.4 si:dkey-22a1.3 snx5 tspan14 tspan15 tspan33a tspan33b tspan5a tspan5b uchl3 vsg1 wnt16 zgc:110329 zgc:113223 zgc:65811 |
| wnt signaling | GO | adgra2 adgra3 aida amer1 amer2 amotl2a ankrd10a ankrd10b ankrd6a ankrd6b ap2m1a ap2m1b apc apc2 axin1 axin2 bambia bambib btk caprin2 ccdc88c csnk1a1 csnk1da csnk1db csnk1e csnk1g1 csnk1g2a csnk1g2b csnk2b csrp1a ctnnb2 ctnnbip1 dact1 dact2 dact3a dixdc1a dixdc1b dkk1a dkk1b dkk2 dkk3a dkk3b dmxl2 dock4a dot1l draxin dub dvl1b dvl2 dvl3a eaf1 eaf2 egf fam132a fam53b fermt2 fgf3 fgf8a foxh1 foxo3b frmd8 frzb fto fzd1 fzd10 fzd2 fzd3a fzd3b fzd4 fzd5 fzd6 fzd7a fzd7b fzd8a fzd8b fzd9a fzd9b gbx1 gnb2l1 gpc3 grk5 hdac1 hs2st1a invs iqgap1 jade1 jun jupa kctd15a kctd15b klhl12 lef1 lrp5 lrp6 lypd6 lzts2a lzts2b med10 mesdc2 metap2b mllt10 mylipa mylipb ndp ndrg2 nipblb nkd1 nkd2a nkd2b nlk1 nlk2 notum1a notum1b notum2 nphp4 nrarpa nrarpb nup62l nxn plpp3 porcn ppp2r3a prdm5 prickle1a psen1 ptk7a rab8b reck rgs17 rgs19 rgs20 rgs3a rhoab rnf14 rnf146 rnf220a rnf220b rock2a rspo1 rspo3 ryk sfrp1a sfrp1b sfrp2 sfrp5 si:ch211-120j21.1 si:ch211-168f7.5 si:ch73-236e11.2 si:dkey-1c11.1 smo sp5l sybu szl ta tax1bp3 tcf7 tcf7l1a tcf7l1b tcf7l2 tlc tmem237b tmem88a tnksa tob1a tollip tpbga tsc2 tspan12 ugdh vangl2 vax2 wif1 wisp3 wls wnt1 wnt10a wnt10b wnt11 wnt11r wnt16 wnt2 wnt2ba wnt2bb wnt3 wnt3a wnt4a wnt4b wnt5a wnt5b wnt6a wnt6b wnt7aa wnt7ba wnt7bb wnt8a wnt8b wnt9a wnt9b wwox zic5 znf703 zranb1a zranb1b |
| Heart function | GO | atp6v0cb cacna1aa cacna1ab cacna1ba cacna1bb cacna1c cacna1da cacna1db cacna1ea cacna1eb cacna1f cacna1fb cacna1g cacna1ha cacna1hb cacna1i cacna1sa cacna1sb cacna2d3 cacna2d4a cacna2d4b cacnb1 cacnb2a cacnb2b cacnb3a cacnb3b cacnb4a cacnb4b cacng1a cacng1b cacng2a cacng2b cacng3a cacng3b cacng4a cacng4b cacng5a cacng5b cacng6b cacng7a cacng7b cacng8a cacng8b ctbp2a ctbp2l dennd5a dennd5b drd2a drd2b drd2l gb:eh507706 gem grm2a grm2b itpr1a itpr1b itpr2 itpr3 loxhd1a loxhd1b mcu orai1a orai1b orai2 pkd1b pkd2 rem1 rem2 rrad ryr1a ryr1b ryr2b ryr3 sgk1 sgk2a sgk2b sgk3 si:ch211-168k15.4 si:ch211-195b13.1 si:ch211-270g19.5 si:ch73-335m24.5 si:ch73-379f7.5 slc24a1 slc24a2 slc24a4a slc24a4b slc30a1a slc30a1b stim1a tmem37 tpcn1 tpcn2 trpc1 trpc2a trpc2b trpc3 trpc4a trpc4b trpc5a trpc5b trpc6a trpc6b trpc7a trpc7b trpm5 trpn1 trpv6 tspan13a tspan13b |
| Heart contraction | GO | adora2aa adra1ba adra1bb adra1d adra2c adra2db adrb2a adrb2b adrbk2 ankhd1 apex1 atp1a2a atp2a1 atp2a2a bag3 bin1b bnip3lb bves cacna1c cacnb1 cald1a cald1b cbfb ccm2 ccm2l chrm2a chrm2b chrm3a cmlc1 cryaba cryabb csrp3 cx39.9 desma desmb dhfr dhrs9 dldh dlst dnd1 erp44 fbxl22 fbxo32 flt1 foxn4 gja3 grk5 hand2 hat hbegfb hel hip hrc hs3st1l1 htr1d htr2a htr2b htr7 hup ik ilk isl1 jam junba kcnh6a kcnk3a kcnk3b kita krit1 leg lims1 lims2 lmna lmod1a lmod1b lmod2b lmod3 lrrc10 lrrc39 lsp1 mcu melk myh6 myl7 mylka mylkb myot nmu ogdha ogdhb parvb pip pkd2 plcg1 plekha7a plk polr2m prdm16 quh rock2b rock2bl ryr1a ryr1b s100a1 scn12aa scn12ab sgcd sgcg sgol1 shox2 shtn1 si:ch211-10p21.1 si:ch211-266g18.10 si:dkey-206m15.8 si:dkey-276l13.4 si:dkey-63b1.1 si:dkey-65b13.9 si:dkeyp-66d7.5 si:dkeyp-68b7.10 si:rp71-17i16.4 sky slc2a12 slc8a1a slc8a4a slmapa slp smpx smyd2a smyd2b smyhc1 smyhc2 sra1 stac3 sth str strip1 strumpellin tacr1a tan taz tbx2b tcap tmod1 tmod2 tmod4 tnnc1a tnnc1b tnnc2 tnni1a tnni1al tnni1b tnni1c tnni1d tnni2a.1 tnni2a.2 tnni2a.3 tnni2a.4 tnni2b. tnni2b.2 tnnt1 tnnt2a tnnt2b tnnt2c tnnt2d tnnt2e tnnt3a tnnt3b tpcn2 tpm4a trdn ttna ttnb unm_tk34b unm_tm96 unm_tr206d unm_ts206 unm_tw212e vcla vdac2 vegfaa viper web zgc:101560 zgc:112242 |
| potassium channel | GO | arpp19a cnga1 cnga3a cnga3b cnga4 cnga5 cngb1a cngb3.1 cngb3.2 cngk hcn1 hcn2b hcn3 hcn4 hcn4l kcna1a kcna2b kcna6a kcnab1a kcnab1b kcnab2a kcnab2b kcnb1 kcnb2 kcnc1a kcnc1b kcnc2 kcnc3a kcnc3b kcnc4 kcnd1 kcnd2 kcnd3 kcne4 kcnf1a kcnf1b kcng1 kcng3 kcng4a kcnh1a kcnh1b kcnh2a kcnh2b kcnh3 kcnh4a kcnh4b kcnh5a kcnh5b kcnh6a kcnh6b kcnh7 kcnh8 kcnip2 kcnj10a kcnj10b kcnj11 kcnj11l kcnj12a kcnj12b kcnj13 kcnj14 kcnj16 kcnj1a.1 kcnj1a.2 kcnj1a.3 kcnj1a.4 kcnj1a.5 kcnj1a.6 kcnj1b kcnj2a kcnj2b kcnj3a kcnj3b kcnj5 kcnj8 kcnj9 kcnk10a kcnk10b kcnk12l kcnk13a kcnk13b kcnk15 kcnk18 kcnk1a kcnk1b kcnk2a kcnk2b kcnk3a kcnk3b kcnk5a kcnk5b kcnk6 kcnk9 kcnma1a kcnma1b kcnmb2 kcnn1a kcnn1b kcnn3 kcnn4 kcnq1 kcnq2a kcnq2b kcnq3 kcnq4 kcnq5a kcnq5b kcns3a kcns3b kcnt1 kcnt2 kcnv2a kcnv2b knca7 rnf207b sgk1 sgk2a sgk2b sgk3 si:ch211-113j13.2 si:ch211-195b13.1 si:ch211-247n2.1 si:ch211-261a10.5 si:ch211-38m6.7 si:ch73-334d15.4 si:dkey-100n10.2 si:dkey-106c17.3 si:dkey-192j17. si:dkey-201i6.2 si:dkey-21e5.1 si:dkey-224b4.1 si:dkey-22i16.2 si:dkey-43k4.5 si:dkey-44k1.5 si:dkey-76p7.5 si:dkeyp-115e12.3 si:rp71-39b20.4 si:rp71-68n21.12 tmem38a tmem38b zgc:162160 |
| Sarcomere | GO | actn2b ankrd1a ankrd1b ankrd2 capn3a capn3b cav3 cbfb cmlc1 csrp3 desma dmd flii hs3st1l1 itgb1b klhl41a klhl41b lrrc39 murca myl7 orai1b rbm24a slc8a1a smyd1a smyd1b smyd2a tfpi2 tln1 tnnt2a tnnt2c tnnt2d tnnt3a tnnt3b ttna ttnb vcla |
